# Development of Timed Release Vaginal Mucosal Cloprostenol for Farrowing Management in Sows

**DOI:** 10.3390/pharmaceutics17091198

**Published:** 2025-09-15

**Authors:** AHM Musleh Uddin, Preechaphon Taechamaeteekul, Kiro R. Petrovski, Padet Tummaruk, Yunmei Song, Sanjay Garg, Roy N. Kirkwood

**Affiliations:** 1 School of Animal and Veterinary Sciences, Adelaide University, Roseworthy Campus, Roseworthy, SA 5371, Australia; ahm.uddin@adelaide.edu.au (A.M.U.); kiro.petrovski@adelaide.edu.au (K.R.P.); 2 Centre for Pharmaceutical Innovation (CPI), College of Health Sciences, Adelaide University, Adelaide, SA 5000, Australia; may.song@unisa.edu.au (Y.S.); sanjay.garg@unisa.edu.au (S.G.); 3 Department of Obstetrics, Gynaecology and Reproduction, Faculty of Veterinary Science, Chulalongkorn University, Bangkok 10330, Thailand; taechamaeteekul.p@gmail.com (P.T.); padet.t@chula.ac.th (P.T.); 4 Davies Livestock Research Centre, Roseworthy Campus, Roseworthy, SA 5371, Australia; 5 Centre of Excellence in Swine Reproduction, Chulalongkorn University, Bangkok 10330, Thailand

**Keywords:** HPLC, quantification, release studies, route of administration, non-invasive, split dose

## Abstract

**Background/Objectives:** Controlling the timing of farrowing to occur during working hours presents an opportunity to improve supervision and reduce piglet neonatal mortality. However, the use of non-therapeutic injectable drugs is often limited in commercial swine production. This study aimed to develop a cloprostenol vaginal mucosal delivery system for induction of farrowing. To achieve this, two vaginal tablets containing cloprostenol were formulated for simultaneous insertion: an immediate-release (IR) tablet and a delayed release (DR) tablet, the latter designed for a 6 h delay before release. **Methods:** In vitro release studies demonstrated that the IR tablet released 100% of the drug within 5 min, while the DR tablet, initiated release after four hours and achieved approximately 80% release at six hours, aligning with the targeted release profile. To evaluate the efficacy of the optimized formulations, an in vivo study was conducted using 121 mixed parity Landrace × Large White sows that were assigned to one of four treatments, control (n = 23) received no treatment; IM (n = 26) received 185 µg of cloprostenol via intramuscular injection; IR (n = 36) received a 100 µg IR tablet by vaginal deposition; and IR + DR (n = 36) received both IR and DR tablets by vaginal deposition, to simulate split-dose delivery. **Results:** Control sows experienced longer (*P* < 0.001) intervals to farrowing compared to those receiving cloprostenol treatments. Additionally, differences (*P* < 0.05) were observed in interval from treatment to farrowing time among the treatments, with the interval for IM sows being shorter than for IR (*P* < 0.001) and IR + DR (*P* = 0.001) sows. **Conclusions:** These findings confirm that the vaginal route offers an alternative, non-invasive, method for farrowing induction in sows, facilitating farrowing supervision during working hours and potentially reducing piglet mortality.

## 1. Introduction

Piglet viability remains a significant challenge for the swine industry, especially as prolificity has risen dramatically in recent years [1]. In commercial farming, it is estimated that 15% to 20% of piglets die within the first few days of life, primarily due to being overlain, starvation, and chilling [2,3]. This issue has become of increasing concern, not only because of its adverse impact on productivity but also due to its implications for animal welfare and the social acceptability of farming practices. Piglet mortality is influenced by various factors including birth weight, environmental conditions, and maternal health [4]. Low birth weight piglets (≤1.1 kg), due to their limited energy reserves and poor thermoregulatory capacity, are particularly vulnerable [1,5]. Prolonged farrowing durations exacerbate these vulnerabilities, increasing the likelihood of hypoxic events and stillbirths [5,6]. Furthermore, farrowing that occur outside working hours pose additional challenges, as unattended deliveries result in higher mortality rates [7,8]. To mitigate these risks, strategies to synchronize farrowing to occur during working hours have been shown to improve neonatal outcomes [7]. However, poorly timed or early inductions can negatively affect piglet birth weight and pre-weaning survival [8].

The efficacy of injection of PGF2α, or analogs, for induction of farrowing is established [9], although large variations in the interval between treatment and parturition are expected. While 80% to 90% of sows may farrow within 36 h of injection, it has been observed that frequently only 50% to 60% of these induced sows farrow during the working day, making them candidates for supervision. It has further been demonstrated that injecting PGF2α into the vulva at 25% to 50% of the manufacturers recommended intramuscular (IM) dose is equally as effective as the full IM dose [3]. The frequency of injections can also affect efficacy, where two injections administered 6 h apart (split-dose) was more effective than a single injection [10,11]. The single dose protocol for the administration of prostaglandins limits the predictability of farrowing, with 50% to 60% of the sows likely farrowing the following working day, compared to >80% following split-dose induction.

A major limitation of current induction protocols within commercial settings lies in the use of injectable treatments, which can induce acute pain and stress in sows [12]. The key challenge arises from managing the frequency and administration of injections, particularly in large-scale operations and, furthermore, issues such as staff forgetting to administer injections or exceeding recommended dosages are of concern. Vaginal mucosal drug deposition offers several advantages as it is a non-invasive, painless and convenient method of drug administration that minimizes the risk of tissue damage; pharmacologically, it facilitates rapid mucosal absorption and reduces hepatic first-pass metabolism [13,14]. We hypothesized that vaginal deposition of a cloprostenol-containing tablet formulated for immediate release would successfully induce farrowing, while deposition of tablets formulated for immediate, as well as a second tablet with a delayed release formulation, will be more effective than the single tablet.

## 2. Materials and Methods

Cloprostenol (purity: 97%) was purchased from BOC Sciences (Shirley, NY, USA); Fructose, Microcrystalline cellulose, Magnesium Stearate, Acetone (HPLC Grade) and Triethyl citrate from Sigma-Aldrich (St Louis, MO, USA); Phosphoric acid from Sigma-Aldrich (Munich, Germany); Lactose from DFE pharma (Goch, Germany); Kiccolate ND 2HS from Asahi Kasei Chemicals (Tokyo, Japan); Aerosil 200 from Evonik Industries (Essen, Germany); Eudragit RL 100 and Eudragit RS 100 were gifted from Evonik Australia Pty Ltd., (Mount Waverly, VIC, Australia); Hydroxypropyl methyl cellulose (HPMC) E4M was purchased from Colorcon Australia (Scoresby, Victoria, Australia); Ethanol (HPLC grade) from Thermo Fisher (Scorseby, VIC, Australia), and Sodium dihydrogen phosphate from AnalaR (Merck, Germany).

### 2.1. HPLC Method for Cloprostenol Quantification

**Figure 1:**
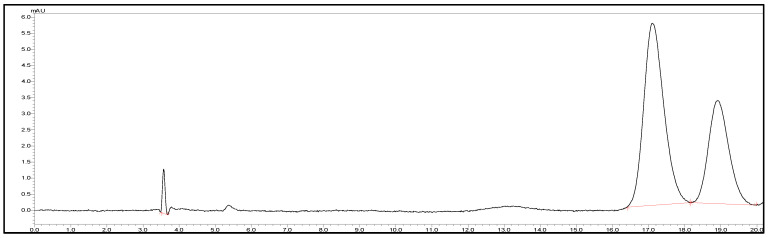
Representative HPLC chromatogram of (±)-cloprostenol showed separation of its two enantiomers.

Chromatographic analysis was performed using a Shimadzu^®^ LC system (Shimadzu Corporation, Kyoto, Japan) with a Lux Cellulose-1 column (Phenomenex, Lane Cove, NSW Australia) set to 20 °C. The mobile phase consisted of an aqueous solution of sodium dihydrogen phosphate dihydrate, adjusted to pH 3.0 with ortho-phosphoric acid [15,16]. This buffer solution was then mixed with acetonitrile in a ratio of 72:28 (*v*/*v*). Flow rate was 0.5 mL/min with an injection volume of 10 μL and signals detected at a wavelength of 210 nm. Linearity range was established across 2.5–50 µg/mL (R^2^ = 0.99), and the retention time was 17.00 min (Figure 1).

### 2.2. Preparation of the Immediate Release (IR) Formulations

Amounts of drug (Cloprostenol-100 µg/tablet), fructose, microcrystalline cellulose (MCC), aerosil and magnesium stearate (MST) were kept at a constant concentration, with varying amount of lactose, croscarmellose sodium (CCS) and crospovidone (CPV), as presented in Table 1. All compositions were weighed using an analytical balance (AB204-S; Mettler Toledo, Switzerland). After appropriate mixing, a direct compression method was used to prepare the tablets.

### 2.3. Preparation of Delayed Release (DR) Coating Formulations

Outer coatings were formulated by combining different polymers in varying weight ratios (Table 2). Eudragit RL 100 and Eudragit RS 100 were dissolved in mixtures of isopropanol (60%) and acetone (40%) while HPMC E4M was dissolved separately in water. The HPMC E4M solution was slowly mixed with the Eudragit RL 100 and Eudragit RL 100 solutions under continuous stirring until a uniform mixture was observed.

For coating purposes, a pan spray coater (Caleva Minnie Coater, Caleva, Dorset, UK) was used with set conditions: fan 75%, agitator 50%, heater 28.00 ± 2 °C, actual temperature 30 °C and pump 4 rpm. The coating process was performed until the tablets attained the expected weight gain [17]. In this process, the coated tablets were weighed intermittently until the expected weight gain was achieved. The mean weight of the coated tablets was then compared to that of the uncoated tablets to determine the actual weight gain, with the results expressed as a percentage [18,19].

### 2.4. Physical Characteristics of Tablets

#### 2.4.1. Weight Variation Test

Ten tablets were randomly selected from each formulation and weighed individually. The mean tablet weight was calculated, and the percentage deviation of each tablet from the mean was determined [20].

#### 2.4.2. Hardness Test

Hardness was determined using a digital force gauge (Electrolab model EH-01P; Cupertino, CA, USA) and recorded the peak breaking point (KPI). The average of ten tablets were tested and reported [16].

#### 2.4.3. Thickness Test

For each formulation, ten tablets were randomly selected and their thickness determined with an external micrometer (0–25 mm; Mitutoyo, Japan) [21]. Results are expressed as mean ± SD.

#### 2.4.4. Friability Test

Friability was assessed by using dual drum friability tester (EF-2 Friabilator USP, Electrolab Pty, Ltd., Goregaon East, Mumbai, India). Approximately 6.50 g of tablets were pre-weighted and placed in friabilator that rotated 100 times and then reweighing after dedusted. Differences between before and after testing was revealed as percentage lost (%) [16].

#### 2.4.5. In Vitro Disintegration Time

Using the method described by Gohel et al. [22] the in vitro disintegration time of immediate-release (IR) tablets was measured with a small-volume Petri-dish setup. Specifically, 10 mL of Milli-Q water at 37 °C was placed in a 10 cm diameter glass Petri dish, the tablet was carefully positioned at the center, and the time to complete disintegration into fine particles was recorded. The measurements were performed in replicates (n = 6) and the results are reported as mean ± SD.

#### 2.4.6. Content Uniformity

For the determination of content uniformity, tablets (n = 10) from selected formulation (CLIR4) were randomly taken and weighed individually. Each tablet was then transferred into a 10 mL volumetric flask containing Milli-Q water and analyzed using the HPLC method [16]. The acceptance value was determined according to the specified formula:

(M − X) + k × s

where

M = reference value,

X = mean of individual contents,

k = acceptability constant (2.4),

s = standard deviation.

#### 2.4.7. Dissolution Test

Release profiles of the IR and DR tablet formulations (coating weight gains: 5, 10, 20 and 30%) were assessed by placing individual tablets into vials containing 10 mL of simulated porcine vaginal fluid (PSVF). These vials were incubated in a shaker set at 37 °C and rotated at 60 rpm. For the DR tablets, 1 mL samples were collected at scheduled time points and replaced with fresh PSVF to maintain a constant volume. The PSVF was prepared following the formulation described by Owen and Katz for human vaginal fluid [23], with the pH adjusted to 7.00 using sodium hydroxide (NaOH). The withdrawn samples were filtered using a 0.45 µm membrane filter and analyzed via high-performance liquid chromatography (HPLC). Each formulation was tested in triplicate, and the average cumulative drug release (%) was plotted over time.

#### 2.4.8. Kinetic Analysis

The release profiles of selected IR and DR formulations during the dissolution studies were analyzed by fitting the cumulative release data to several mathematical kinetic models [24]. The quality of fit for each model was determined by calculating the adjusted coefficient of determination (R^2^-adjusted), the Akaike Information Criterion (AIC), and the model selection criterion (MSC). Kinetic modeling was conducted using DDSolver (an Excel-based add-in program).

### 2.5. Fourier Transform Infrared Spectroscopy (FTIR)

The character of the pure drug, drug with excipients and blank formulation (without pure drug) was identified by the FTIR spectrometer (Bruker, Billerica, MA, USA). The spectra were recorded at room temperature with a wavenumber range of 4000 to 400 cm^−1^ in transmittance mode, using each analysis comprising four scans at a resolution of 4.0 cm^−1^ [25].

### 2.6. Differential Scanning Calorimetry (DSC)

DSC measurements for pure drug, blank formulation (without active ingredient), physical mixture (drug with all excipients), and individual excipients including magnesium stearate (MST), microcrystalline cellulose (MCC), lactose, fructose, croscarmellose sodium (CCS), and aerosil 200 were determined in Discovery DSC 2920 (TA Instruments, New Castle, DE, USA) calibrated with an indium standard. Samples weighing 4.00 ± 0.50 mg were placed in aluminum pans and thermal profiles was recorded by heating the samples from 10 to 300 °C at a rate of 10 °C/min under continuous nitrogen gas flow [25].

### 2.7. Scanning Electron Microscopy (SEM)

Surface morphology of the coated tablets analyzed at Adelaide Microscopy, South Australia, using a FEI Quanta 450 FEG environmental scanning electron microscope (ESEM). This high-resolution field emission SEM was employed to examine the surface topography, detect structural imperfections such as cracks and pores, and assess coating uniformity. Additionally, backscattered electron imaging and elemental analysis were conducted using a silicon drift detector (SDD) with energy dispersive spectroscopy (EDS) system.

Samples were mounted on aluminum stubs using double-sided carbon tape and sputter-coated with a thin layer of platinum to improve conductivity. SEM imaging was performed under high-vacuum conditions with an accelerating voltage of 20 kilovolt (kV). The data were acquired using element normalization, with SEC table set to default and standardless mode for EDS analysis. Images were captured at multiple magnifications to evaluate the quality of the polymeric coating and identify any surface defects that could impact delayed drug release performance.

### 2.8. In Vivo Experiment

Following in vitro tablet assessment, the chosen tablet formulations were tested in vivo in mixed parity (1 to 7; mean 3.30 ± 1.90), Landrace x Large White sows housed on a commercial 5000-sow farm in northern Thailand, with approval of The University of Adelaide Animal Ethics Committee (Approval No. S-2023-110) and conducted in accordance with the Australian Code for the Care and Use of Animals for Scientific Purposes (8th edn, 2013) and the Animal Welfare Act (South Australia).

At 109.0 ± 2.0 days of gestation, 121 sows were moved into individual farrowing crates in a room with an evaporative cooling system. The crate swing-gates remained closed until 4 days post-partum. From entry to the farrowing house until farrowing, sows were fed 3.0–3.5 kg per sow of a lactation diet formulated to contain 17.2% crude protein, 13.8 MJ ME/kg, 4.3% fiber, and 1.1% lysine. Feed, provided in pellet form, was distributed 4 times daily at 06:00 am, 10:00 am, 01:00 pm, and 04:00 pm. Water was continuously available through drinking nipples.

Sows were assigned, by parity, to one of 4 treatments, with all treatments applied at 08:00 h on day 113 gestation:

- Control (n = 23) received no induction treatment.

- IM (n = 26) received an intramuscular injection of 185 µg cloprostenol (2 mL Planate^®^, Merck, NJ, USA).

- IR (n = 36) received a single IR tablet containing 100 µg cloprostenol.

- IR + DR (n = 36) received a 100 µg IR tablet and a second 100 µg cloprostenol tablet coated to delay the release for 4 to 6 h.

The interval from treatment to delivery of the first piglet was recorded.

### 2.9. Statistics

All statistical analyses were conducted using SAS version 9.4 (Statistical Analysis Software, Cary, NC, USA). Data were bootstrapped at a root of 24 using PROC SURVEYSELECT, yielding a total of 621 sows level observations for analysis. The quality of the bootstrapped data was cross-checked using PROC MEANS, confirming consistency within the second decimal point compared to the original data.

Correlations between the time of farrowing and experimental groups were tested using PROC CORR with the output being the Pearson’s correlation coefficient and the respective 95% confidence intervals. Correlation was very high if *r* ≥ 0.90, high if *r* = 0.70 to 0.89, moderate if *r* = 0.50 to 0.69, low if *r* = 0.30 to 0.49, and negligible if *r* < 0.3 [26].

The effect of route of administration of cloprostenol on the interval from treatment to farrowing onset were estimated using a Mixed model in PROC MIXED. The outputs were the least-square means, their respective standard errors, and differences between the least-square means. A *P*-value of <0.05 was considered statistically significant.

## 3. Results

### 3.1. Physical Properties

Physical characteristics of eight tablet formulations (CLIR1 to CLIR8) including weight uniformity, hardness, thickness, friability, and disintegration time were tested (Table 3). All the formulations exhibited values within acceptable ranges of solid dosage forms. Among them, CLIR4 was identified as the most suitable formulation with the least disintegration time (0.62 ± 0.04 min), indicating its promise for rapid drug release. The formulation also exhibited acceptable mechanical strength and physical integrity.

### 3.2. Drug Uniformity Test

Twelve tablets from each of 3 batches of CLIR4 were assessed for cloprostenol content uniformity (Table 4). The Acceptance values were determined per the United States Pharmacopeia (USP) specified limit for solid dosage forms (L1 ≤ 15) [20].

### 3.3. In Vitro Release Studies

**Figure 2:**
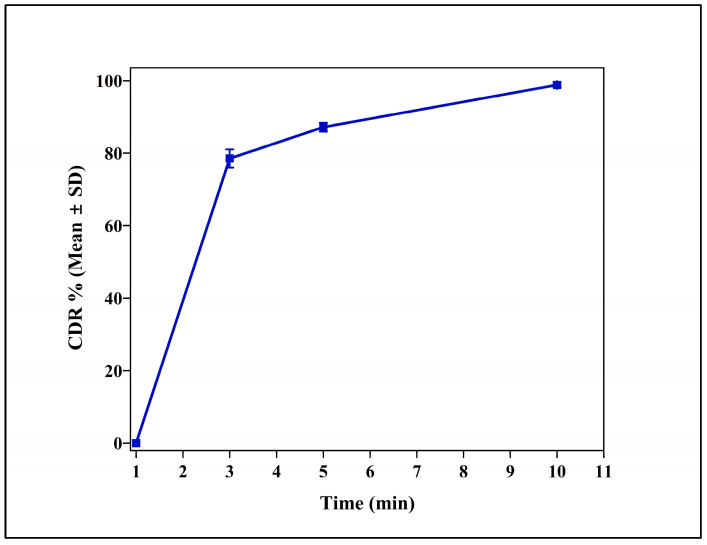
Cumulative drug release (CDR %) profile of the immediate release (IR) tablet formulation (CLIR4) over a 10 min period. The data are expressed as mean ± standard deviation (SD) (n = 3).

IR tablets disintegrated within 5 min of contact with SPVF, with complete release of cloprostenol within 10 min (Figure 2). Croscarmellose sodium (Kiccolate ND-2HS) played a crucial role for the achievements of the quick disintegration and release of drugs [27].

### 3.4. Delayed Release Formulation

**Figure 3:**
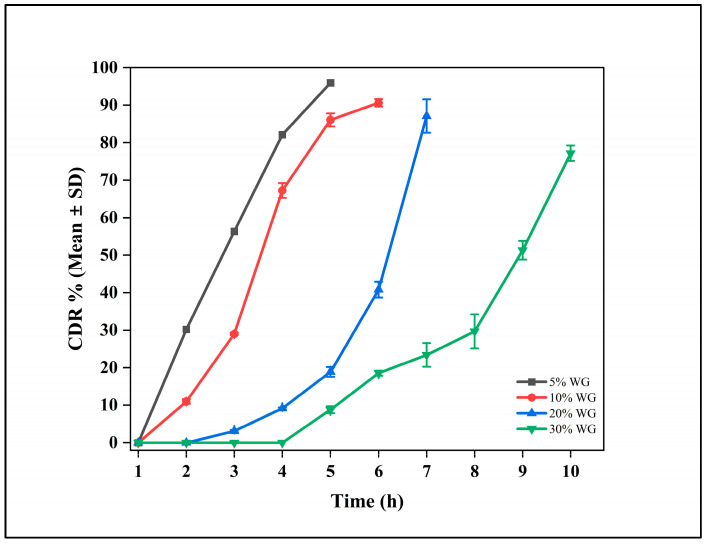
Cumulative drug release (CDR %) profiles of delayed release (DR) tablet (CLDR8) with varying coating weight gains (WG) of 5%, 10%, 20%, and 30%. The data are expressed as mean ± standard deviation (SD) (n = 3).

Among all the dissolution profiles, only the outer coating formulation containing Eudragit RS 100, Eudragit RL 100, and HPMC E4M in a 2:1:1 ratio exhibited a remarkable burst release after 5 h, achieving approximately 80% drug release within 6 h. This release profile aligned with our target for in vitro studies (Figure 3).

### 3.5. Fourier Transform Infrared Spectroscopy (FTIR)

**Figure 4:**
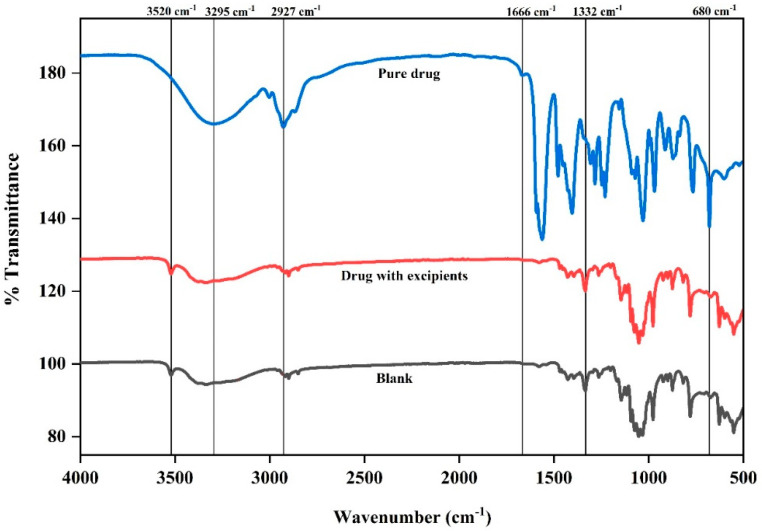
Fourier transform infrared (FTIR) spectra of the pure drug, the drug with excipients (physical mixture), and the blank formulation (without active ingredient). Characteristic peaks are labeled to identify potential interactions between the drug and excipients.

The FTIR spectra for the pure drug, powder mix (drug with excipients), and blank formulation are presented in Figure 4. In the functional group region, the sharp peak observed at 1666 cm^−1^ in the pure drug confirmed the presence of C=O stretching vibrations, a distinguishing feature of the carboxylic acid group. However, in the powder mix, that peak became slightly broadened, suggesting weak interactions between pure drug and others excipients through hydrogen bonding or van der Waals forces [28]. In addition, the absence of a peak in that region in the blank sample confirmed the absence of pure drug.

At 3295 cm^−1^ the pure drug demonstrated a deeper and broader nadir (stretching peak), supporting the presence of intra-molecular hydrogen bonding within the pure drug structure. On the other hand, this peak exhibited further broadening, indicating the formation of inter-molecular hydrogen bonding between cloprostenol and hydrophilic excipients such as MCC and CCS in the powder mix. A similar O-H stretching band was also evident in the blank formulation, primarily attributed to the contributions of hydrophilic excipients like MCC, CCS, and lactose.

The deeper and broader nadir at ~2927 cm^−1^ (C-H stretching peak) observed in the pure drug signifies a highly uniform molecular environment with minimal external interactions, ensuring a consistent and well-defined vibrational state (Figure 3). Conversely, in powder mix, this peak appeared narrower, which can be attributed to the masking the pure drug peak due to excipient interactions. In contrast, the blank sample exhibited a similar C-H stretching peak, primarily arising from the contributions of excipients.

In fingerprint region, the sharp and well-defined peak at 680 cm^−1^ was representative of the C-Cl stretching vibrations for the pure drug. However, this peak was broadened with lower intensity in the powder mix, suggesting that the interactions between cloprostenol and excipients are weak due to van der Waals forces or slight hydrogen bonding without affecting the chemical structure of pure drug. Meanwhile, the absence of this peak in the blank formulation confirmed that it was specific to cloprostenol. Furthermore, in the presence of aerosil, the Si-O stretching peak (~1332 cm^−1^) was present in the powder mix and blank formulation but not in the pure drug.

In the blank formulation, the 3520 cm^−1^ peak remains prominent and slightly broader compared to fructose alone. This is due to contributions from multiple excipients, such as MCC, CCS, and fructose, which contain hydroxyl groups. The peak confirms that fructose is the primary contributor in this wavenumber region (Supplementary Figure S1).

These results confirm that there were no notable chemical interactions between cloprostenol and the excipients. The absence of cloprostenol specific peaks in the blank sample shows that the excipients MCC, CCS, lactose, fructose, and aerosil 200 are compatible with the drug.

### 3.6. Differential Scanning Calorimetry (DSC)

**Figure 5:**
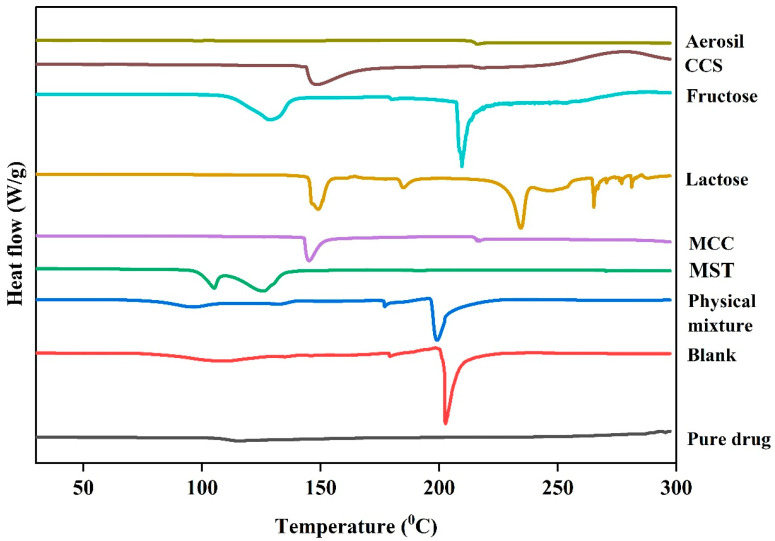
Differential scanning calorimetry (DSC) thermograms of the pure drug, blank formulation (without active ingredient), physical mixture (drug with all excipients), and individual excipients including magnesium stearate (MST), microcrystalline cellulose (MCC), lactose, fructose, croscarmellose sodium (CCS), and aerosil 200.

DSC analysis was performed on pure cloprostenol, the physical mixture, blank formulations, and individual excipients and are presented in Figure 5 and Supplementary Figure S1. The DSC thermogram of pure cloprostenol exhibited a sharp endothermic peak at 115 °C, corresponding to the melting point of the drug crystals, confirming its highly crystalline nature. Notably, in physical mixtures containing very low drug concentrations, the 115 °C melting peak of cloprostenol was significantly diminished, and no distinct melting peak was observed. This suggests that the crystalline structure of cloprostenol was altered, and the drug was likely dispersed in an amorphous form within the excipient matrix.

In the blank formulations, no thermal events corresponding to the cloprostenol melting point were observed. However, a pronounced thermal event at 200 °C was detected, which is attributed to the melting or decomposition of excipients. Similarly, both the physical mixture and blank formulations displayed exothermic peaks above 250 °C, indicative of the thermal decomposition of cloprostenol and excipients.

Additionally, a thermal event was observed around 100 °C in both the physical mixture and blank formulations. This event is likely due to moisture loss or dehydration of hygroscopic excipients, as it was absent in the pure cloprostenol thermogram.

### 3.7. Scanning Electron Microscopy (SEM)

**Figure 6:**
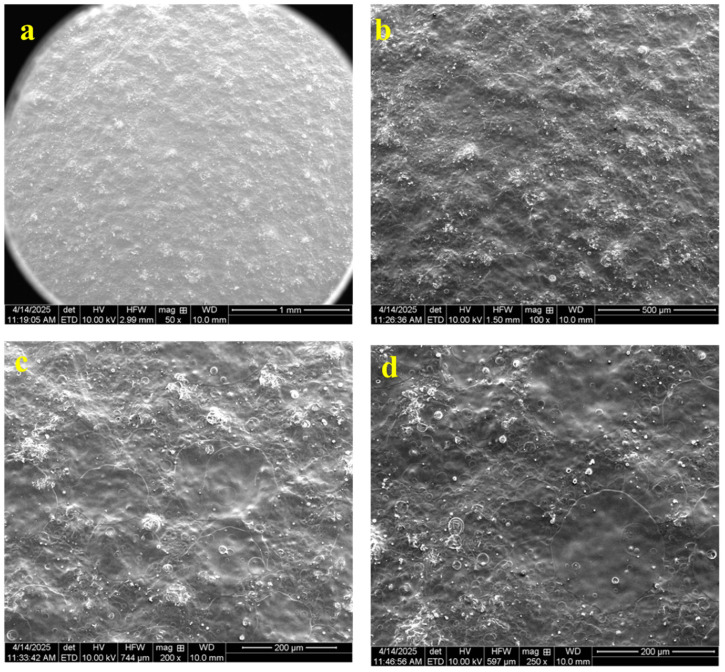
Scanning electron microscopy (SEM) images illustrated the surface morphology of coated cloprostenol tablets. Panels a–d display different magnifications: (**a**) 50×, (**b**) 100×, (**c**) 200×, and (**d**) 250×.

SEM analysis revealed that the coated tablet exhibited a generally smooth and uniform surface morphology (Figure 6). The coating appeared as a continuous film encapsulating the tablet, with no visible cracks, fractures, or discontinuities. The surface was largely featureless and even, suggesting effective coalescence of the polymer blend into an intact film. No apparent defects or exposed areas of the tablet were observed, indicating high coating integrity and complete coverage.

### 3.8. Mathematical Model for Drug Release Mechanism

In this study, the drug release mechanisms of IR and DR tablets were evaluated using various mathematical models. The results provide insights into the underlying processes governing drug release from these formulations.

### 3.9. Immediate Release (IR) Tablets

IR tablets are designed to release the drug rapidly after administration in the intravaginal route to facilitate the maximum permeation through the vaginal mucosa. From Table 4, best fitting models for the IR tablets are the Makoid–Banakar and Peppas–Sahlin models as interpreted by high R^2^-adjusted values (0.9958 for both models), low AIC values (2.3296 and 2.3827, respectively), and high MSC values (5.0763 and 5.0630, respectively) [16]. These models suggest that cloprostenol release from IR tablets is regulated by a combination of mechanisms such as burst release, diffusion (cloprostenol diffuses through the matrix), erosion (matrix undergoes gradually erosion and releasing the drug) and relaxation (polymer chains relax and release the drug) [29,30]. The rapid release is facilitated by the combined effects of diffusion and erosion, ensuring an immediate and complete release of the drug.

### 3.10. Delayed Release (DR) Tablets

Cloprostenol DR tablets are designed to release the drug after a lag time in the intravaginal space to achieve the target outcomes in the farrowing parameters. Analysis of the mathematical kinetics (Table 5) revealed that the Makoid–Banakar, Peppas–Sahlin and Korsmeyer–Peppas models showed the best fit for the DR tablet, as indicated by high R^2^-adjusted values (0.9995, 0.9994 and 0.9980, respectively), low AIC values (11.3106, 12.3105 and 20.3011, respectively), and high MSC values (6.9436, 6.8008 and 5.6593, respectively). These models indicated that cloprostenol release from coated DR tablets is mainly regulated by mechanism including diffusion (drug diffuses through a polymeric coating) and relaxation (polymer swells or relaxes, facilitating the drug release) [29,30,31]. The Korsmeyer-Peppas model describes the drug released from the polymer-based systems [32,33]. The ideal fit of this model indicates that the drug release from coated DR tablets is diffusion-controlled, with the polymer matrix playing a key role in regulating the release rate over time.

### 3.11. In Vivo Experiment

**Figure 7:**
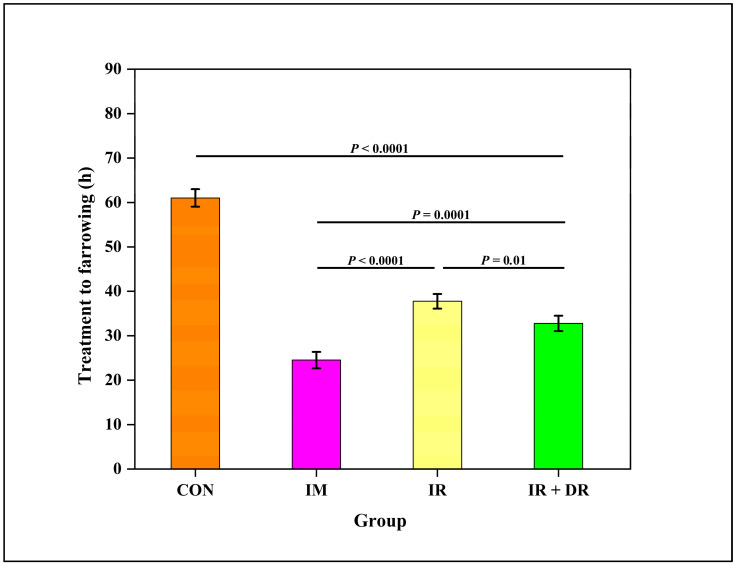
Effect of cloprostenol administration methods on the interval from treatment to farrowing (hours) in sows. The groups include CON (Control), no treatment; IM (Intramuscular injection), IR (Immediate release tablet), IR + DR (immediate and delayed release tablets). The data are presented as Least square mean ± standard error of the mean (SEM), with whiskers representing SEM.

The interval to farrowing of sows in the control group (61.1 ± 4.7 h) was longer than the treatment to farrowing interval for the IM (25.0 ± 4.4 h), IR (41.0 ± 3.7 h), and IR + DR (37.5 ± 3.7 h) sows. However, the interval in the IM group was shorter than in the IR and DR sows (Figure 7). Interestingly, a difference (*P* ˂ 0.05) in farrowing time between the IR and IR plus DR groups was evident.

**Figure 8:**
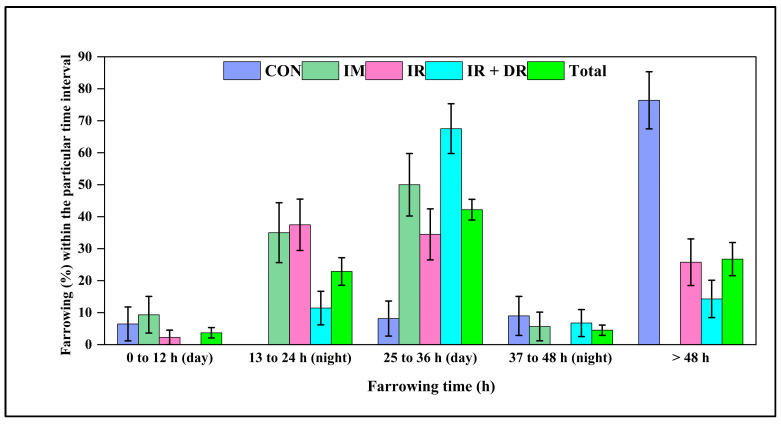
Farrowing percentage among different groups of sows following cloprostenol administration. The groups include CON (Control), no treatment, IM (Intramuscular injection), IR (immediate release tablet), and IR + DR (immediate and delayed release tablets). Farrowing (%) is categorized into 0–12 h (day), 13–24 h (night), 25–36 h (day), 37–48 h (night), and >48 h post-treatment. Total represents the overall farrowing across all treatment groups combined. The data are presented as the percentage of sows farrowing within each time interval ± standard error of the mean (SEM).

As shown in Figure 8, almost 80% of control sows farrowed more than 48 h after treatment. The IM sows exhibited a faster farrowing response, with most sows farrowing within 13–36 h after treatment. The proportion of sows farrowing beyond 36 h was minimal, demonstrating the effectiveness of IM administration in reducing farrowing time. The IR group showed a more even distribution of farrowing times, with the majority occurring within 13–36 h. Compared to IR, more IR + DR sows farrowed during the subsequent working day 25–36 h. There were no differences in total born litter size or numbers stillborn (Table 6).

## 4. Discussion

This study aimed to develop and evaluate a novel intravaginal cloprostenol formulation, which includes IR and DR tablets for the induction of farrowing in sows. The DR tablet demonstrated controlled release profiles depending on the ratio of Eudragit RS and RL polymers and the coating weight gain, confirming the system’s potential for time-specific drug delivery. In vivo, the double tablet, simulating split-dose administration effectively induced farrowing in a similar timeframe to the intramuscular injection, with over two-thirds of treated sows farrowing the next day during working hours. This approach improved farrowing supervision, enhanced piglet care, and may serve as a practical alternative to intramuscular injection. Notably, the intravaginal system was easy to administer and reduced handling time and effort for farm staff, offering practical advantages for on-farm application.

From our in vitro data, it was evident that CCS exhibited a higher disintegration efficiency than CPV due to its dual disintegration mechanism, which combined swelling and wicking, promoting rapid tablet breakdown [34]. Unlike CPV, which primarily relies on capillary action, CCS demonstrated high swelling capacity, allowing it to create internal stress within the tablet matrix and facilitate faster drug release.

The combination of CCS and lactose has proven particularly effective in pharmaceutical formulations, as lactose serves as a highly water-soluble filler enhancing the disintegration efficiency of CCS [34]. Since lactose rapidly dissolves upon contact with water, it allowed CCS to swell more effectively and exert its disintegration force. Moreover, lactose helped create soft, porous compacts, further improving water penetration and overall tablet disintegration [35]. This synergistic effect makes CCS and lactose one of the best combinations for fast-acting, immediate-release formulations.

The drug release profiles of DR tablets demonstrated that the polymer ratio, particularly the proportion of Eudragit RS 100, played a crucial role in controlling the drug release profile in aqueous media due to its low permeability. Eudragit polymers, when used as coating materials, effectively sustain drug release over time [36,37]. Our study observed that the mixture of Eudragit RS 100, Eudragit RL 100, and HPMC E4M, combined with controlled weight gain of the coating, allowed precise modulation of drug release over the intended time. Our findings were further supported by the proposed mechanism of drug release from dosage forms coated with Eudragit RS and RL mixtures, which occurred through controlled fluid permeation into the tablet core, followed by dissolution and outward diffusion of the active substance [38].

For the in vivo study, the present data reported that depositing approximately half the intramuscular dose (100 µg/tablet) was capable of successfully inducing farrowing in about 80% of sows within 36 h, supporting previous findings [3,39]. The minimum effective dose of cloprostenol required for complete functional luteolysis and a prompt farrowing has not yet been determined. Our intravaginal tablets revealed a comparable farrowing induction response to IM injection regardless of the dose. Interestingly, the simultaneous deposition of IR and DR tablets resulted in two-thirds of sows farrowing the following day during working hours, enhancing farrowing supervision, potentially may lead to reduced neonatal mortality, improved colostrum intake, and timely fostering when required. However, this response was underpinned by more IR sows farrowing earlier (13 to 24 h), suggesting that our modern sows may be more sensitive to cloprostenol action than those described in earlier studies.

## 5. Conclusions

Intravaginal cloprostenol tablets offer a practical, needle-free alternative to injectable protocols for inducing farrowing in sows. The immediate release formulation exhibited complete drug release within five minutes. In contrast, the delayed release tablet-initiated release after four hours and reached 80% release at six hours, demonstrating strong consistency with the targeted release profile. Controlled drug release was effectively achieved through a blend of Eudragit RS 100, Eudragit RL 100, and HPMC E4M, ensuring expected performance. The newly developed IR and DR tablets, when administered as a split dose of cloprostenol via the vaginal route, successfully synchronized farrowing within working hours, thereby facilitating timely assistance and improving both animal welfare and operational efficiency. Further research is warranted to optimize dosing strategies and evaluate efficacy across larger and more diverse sow populations, supporting broader adoption in commercial swine production systems. In particular, targeting sow herds demonstrating a limited response to a single treatment would prove valuable.

## Figures and Tables

**Table 1:** Composition of immediate-release (IR) tablet formulations (CLIR1 to CLIR8). The total tablet weight was maintained at 120.0 mg per tablet across all formulations.

Compositions	List of Formulation							
	CLIR1	CLIR2	CLIR3	CLIR4	CLIR5	CLIR6	CLIR7	CLIR8
**Fructose (mg)**	64.0	64.0	64.0	64.0	64.0	64.0	64.0	64.0
**Lactose (mg)**	24.0	21.5	18.5	16.5	24.0	21.5	18.5	16.5
**MCC (mg)**	24.0	24.0	24.0	24.0	24.0	24.0	24.0	24.0
**CCS (mg)**	2.5	5.0	8.0	10.0	0	0	0	0
**CPV (mg)**	0	0	0	0	2.5	5.0	8.0	10.0
**Aerosil 200 (mg)**	2.5	2.5	2.5	2.5	2.5	2.5	2.5	2.5
**MST (mg)**	3.0	3.0	3.0	3.0	3.0	3.0	3.0	3.0
**Weight per tablet (mg/tab)**	120.0	120.0	120.0	120.0	120.0	120.0	120.0	120.0

**Table 2:** Polymer composition and ratios used for outer coating in delayed release (DR) tablet formulations (CLDR1 to CLDR8). The polymer ratios are expressed as percentages of the total coating formulation.

Formulations No.	List of Polymers		
	Eudragit RS 100 (%)	Eudragit RL 100 (%)	HPMC E4M (%)
CLDR1	–	70	30
CLDR2	–	50	50
CLDR3	–	30	70
CLDR4	70	–	30
CLDR5	50	–	50
CLDR6	30	–	70
CLDR7	55	22.5	22.5
CLDR8	50	25	25

**Table 3:** Physical properties of different Immediate release (IR) tablet formulations (CLIR1 to CLIR8). Each formulation was evaluated for weight variation, hardness, thickness, friability, and disintegration time. Results are presented as mean ± standard deviation (SD), where applicable.

Formulations	Weight Variations (mg)	Hardness (kgf)	Thickness (mm)	Friability (%)	Disintegration Time (min)
CLIR1	113.16 ± 2.34	6.40 ± 0.11	3.01 ± 0.05	0.33	3.50 ± 0.04
CLIR2	122.60 ± 2.76	5.92 ± 0.18	3.24 ± 0.08	0.37	2.10 ± 0.04
CLIR3	124.83 ± 6.10	6.01 ± 0.10	3.45 ± 0.15	0.36	1.27 ± 0.02
CLIR4	121.87 ± 2.46	5.75 ± 0.09	3.20 ± 0.04	0.35	0.62 ± 0.04
CLIR5	130.24 ± 2.02	5.95 ± 0.09	3.51 ± 0.05	0.62	5.29 ± 0.08
CLIR6	131.51 ± 1.99	5.98 ± 0.08	3.56 ± 0.04	0.57	4.26 ± 0.02
CLIR7	123.71 ± 2.21	5.87 ± 0.08	3.22 ± 0.03	0.73	2.94 ± 0.17
CLIR8	124.37 ± 2.72	5.90 ± 0.11	3.29 ± 0.07	0.73	1.37 ± 0.02

**Table 4:** Uniformity test of selected Immediate release (IR) tablet formulation (CLIR4) across three independent batches (n = 12 per batch). Results are expressed as mean ± SD, with Acceptance Value (L1 ≤ 15).

Formulations	Batch No	Desired Drug Amount (µg)	Average Drug Amount (µg)	Acceptance Value (L1 ≤ 15)
CL55IR4	CL55IR4B1	100	99.75 ± 0.12	0.33
	CL55IR4B2	100	100.98 ± 2.09	5.44
	CL55IR4B3	100	100.03 ± 0.90	2.17

**Table 5:** Model parameters to describe the in vitro release kinetics of selected immediate release (IR) and delayed release (DR) intravaginal cloprostenol tablets. The models evaluated include Zero-order, First-order, Higuchi, Hixson–Crowell, Hopfenberg, Makoid–Banakar, Peppas–Sahlin, Weibull, and Korsmeyer–Peppas. The parameters reported are the adjusted coefficient of determination (R^2^-adjusted), Akaike Information Criterion (AIC), and Model Selection Criterion (MSC).

Model Name	Parameter	IR Tablet	DR Tablet
Zero order	R^2^-adjusted	–19.22	0.56
	AIC	36.66	57.41
	MSC	–3.50	0.35
First order	R^2^-adjusted	0.94	0.48
	AIC	13.33	58.55
	MSC	2.32	0.19
Higuchi	R^2^-adjusted	–3.87	0.32
	AIC	30.96	60.45
	MSC	–2.08	−0.07
Hixson–Crowell	R^2^-adjusted	–0.06	0.50
	AIC	24.89	58.23
	MSC	–0.56	0.24
Hopfenberg	R^2^-adjusted	0.91	0.96
	AIC	15.33	40.18
	MSC	1.82	2.81
Makoid–Banakar	R^2^-adjusted	0.99	0.99
	AIC	2.32	11.31
	MSC	5.07	6.943
Peppas–Sahlin	R^2^-adjusted	0.99	0.99
	AIC	2.38	12.31
	MSC	5.06	6.80
Weibull	R^2^-adjusted	0.97	0.98
	AIC	9.05	35.56
	MSC	3.39	3.47
Korsmeyer–Peppas	R^2^-adjusted	0.88	0.99
	AIC	16.48	20.30
	MSC	1.53	5.65

**Table 6:** Effect of cloprostenol administration via intramuscular injection (IM) and single (IR) or double (IR + DR) intravaginal tablets on litter characteristics in sows. Results are presented as mean ± standard error of the mean for the total number of piglets born and number of stillbirths per litter.

Parameter	Control	IM	IR	IR + DR
Total born	13.55 ± 0.79	13.09 ± 0.71	14.72 ± 0.65	13.22 ± 0.69
Stillbirth	0.39 ± 0.27	0.40 ± 0.24	0.88 ± 0.21	0.37 ± 0.22

## Data Availability

The data presented in this study are available on request from the corresponding author.

## Supplementary Materials

The following supporting information can be downloaded at: https://www.mdpi.com/article/10.3390/pharmaceutics17091198/s1, Supplementary Figure S1: Differential scanning calorimetry (DSC) thermograms of the Cloprostenol (A), Blank formulation without drug (B), Physical mixture with drug (C), Magnesium stearate (D), Microcrystalline cellulose (E), Lactose (F), Fructose (G), Croscarmellose sodium (H) & Aerosil 200 (I).
