# Supplementary material for: Development of Timed Release Vaginal Mucosal Cloprostenol for Farrowing Management in Sows
Source: Pharmaceutics. 2025 Sep 15;17(9):1198. doi: 10.3390/pharmaceutics17091198 (PMC12473592; doi:10.3390/pharmaceutics17091198)
Supplement: Supplementary file 1 [file pharmaceutics-17-01198-s001.zip › pharmaceutics-3846281-supplementary.pdf]

Article

Development of Timed Release Vaginal Mucosal Cloprostenol for Farrowing Management in Sows

AHM Musleh Uddin [1,2], Preechaphon Taechamaeteekul [3], Kiro R. Petrovski [1,4,], Padet Tummaruk [3,5], Yunmei Song [2], Sanjay Garg [2] and Roy N. Kirkwood [1,*]

[1] School of Animal and Veterinary Sciences, Adelaide University, Roseworthy Campus, Roseworthy, SA 5371, Australia
[2] Centre for Pharmaceutical Innovation (CPI), College of Health Sciences, Adelaide University, SA 5000, Australia
[3] Department of Obstetrics, Gynaecology and Reproduction, Faculty of Veterinary Science, Chulalongkorn University, Bangkok, 10330, Thailand
[4] Davies Livestock Research Centre, Roseworthy Campus, Roseworthy, SA 5371, Australia
[5] Centre of Excellence in Swine Reproduction, Chulalongkorn University, Bangkok, 10330, Thailand
* Correspondence: roy.kirkwood@adelaide.edu.au

Supplementary Figure S1: Differential scanning calorimetry (DSC) thermograms of the Cloprostenol (A), Blank formulation without drug (B), Physical mixture with drug (C), Magnesium stearate (D), Microcrystalline cellulose (E), Lactose (F), Fructose (G), Croscarmellose sodium (H) & Aerosil 200 (I).

A

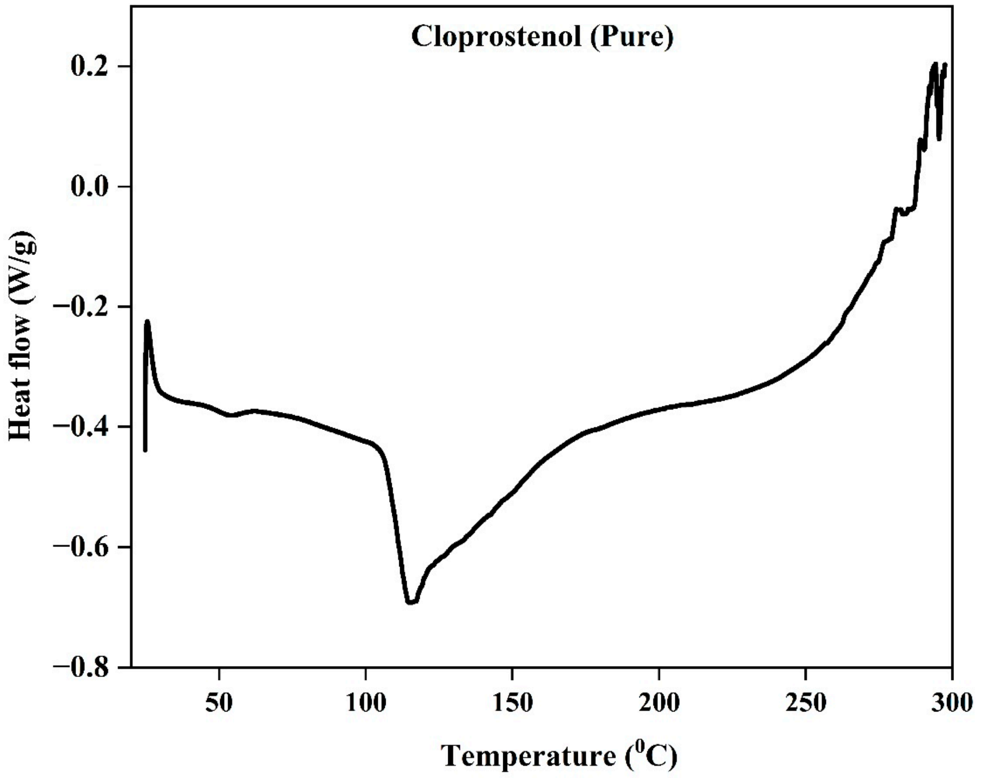

B

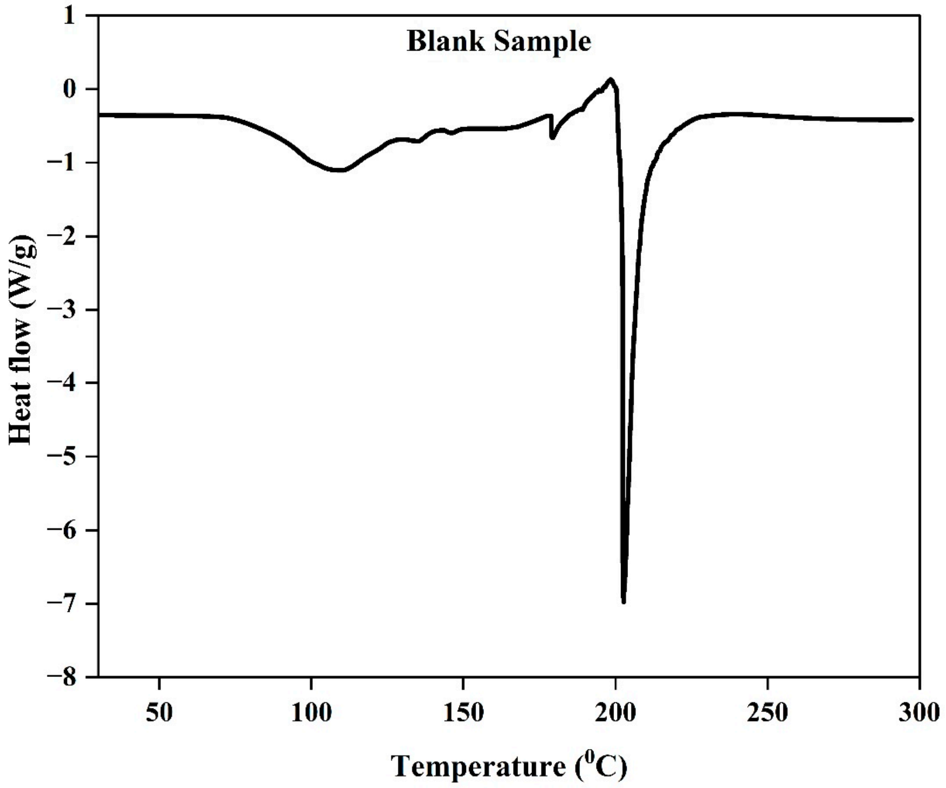
Blank Sample
Heat flow (W/g)
1
0
−1
−2
−3
−4
−5
−6
−7
−8
50
100
150
200
250
300
Temperature (^{0}C)

C

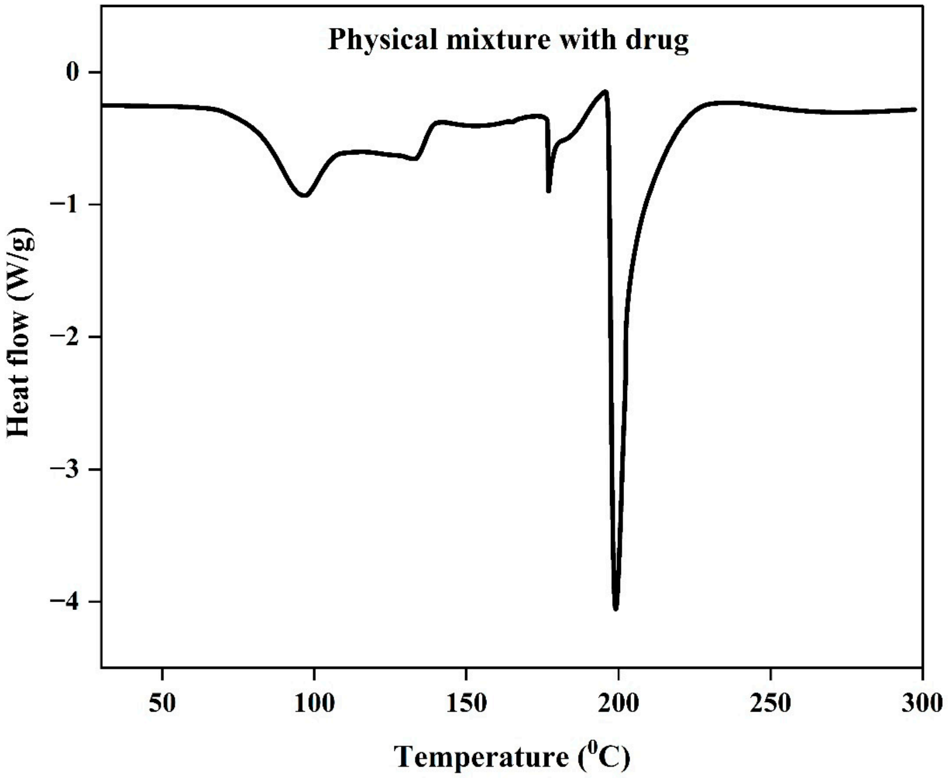
Physical mixture with drug
Heat flow (W/g)
0
−1
−2
−3
−4
50
100
150
200
250
300
Temperature (^{0}C)

D

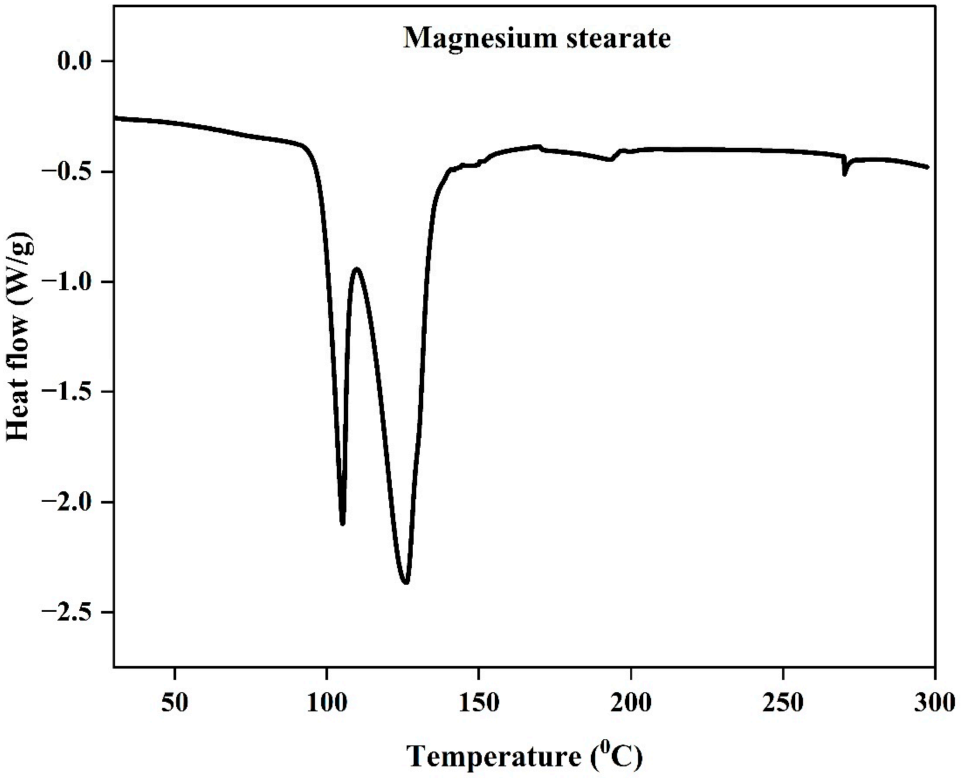
Magnesium stearate
Heat flow (W/g)
0.0
−0.5
−1.0
−1.5
−2.0
−2.5
50
100
150
200
250
300
Temperature (0C)

E

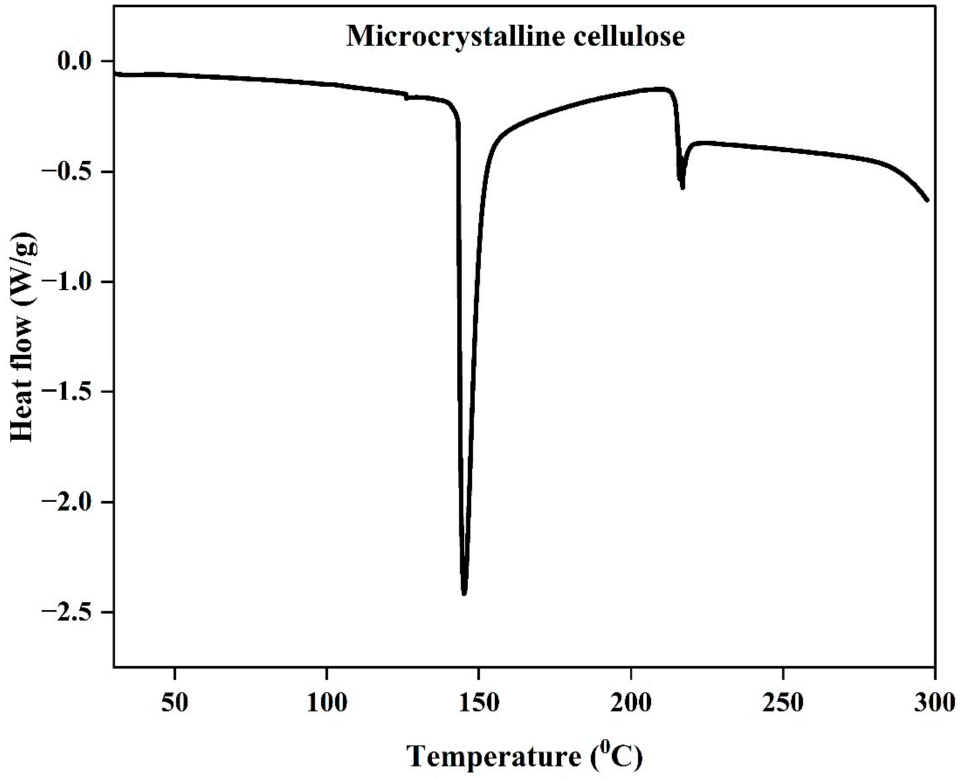
Microcrystalline cellulose
0.0
−0.5
−1.0
−1.5
−2.0
−2.5
Heat flow (W/g)
50
100
150
200
250
300
Temperature (0C)

G

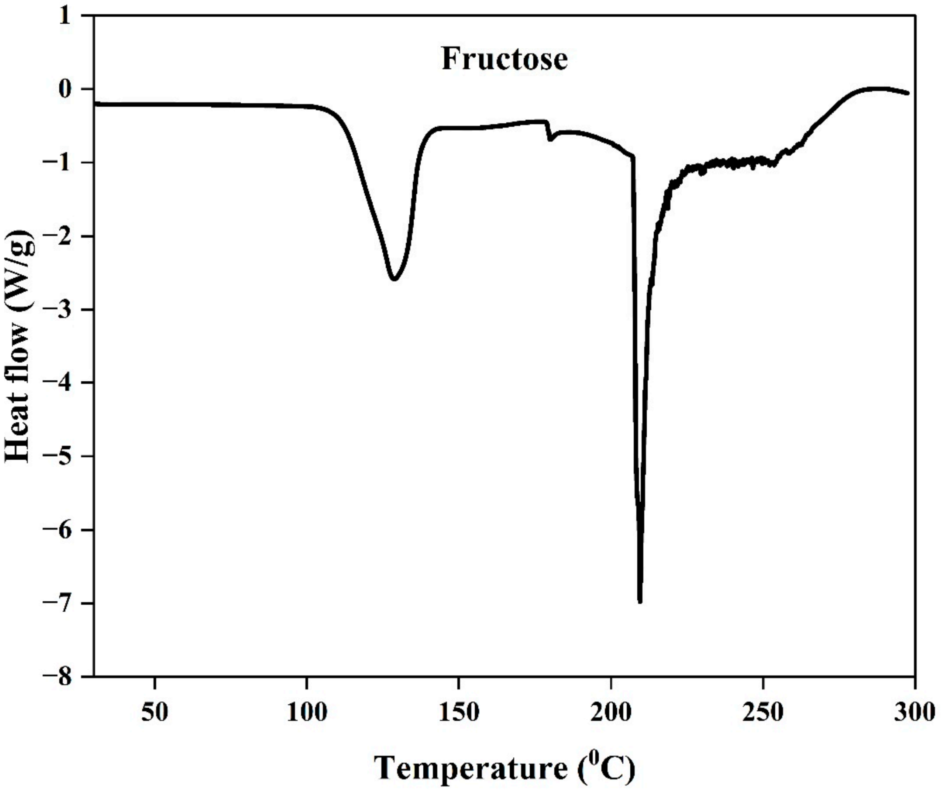
Fructose
Heat flow (W/g)
1
0
−1
−2
−3
−4
−5
−6
−7
−8
50
100
150
200
250
300
Temperature (0C)

H

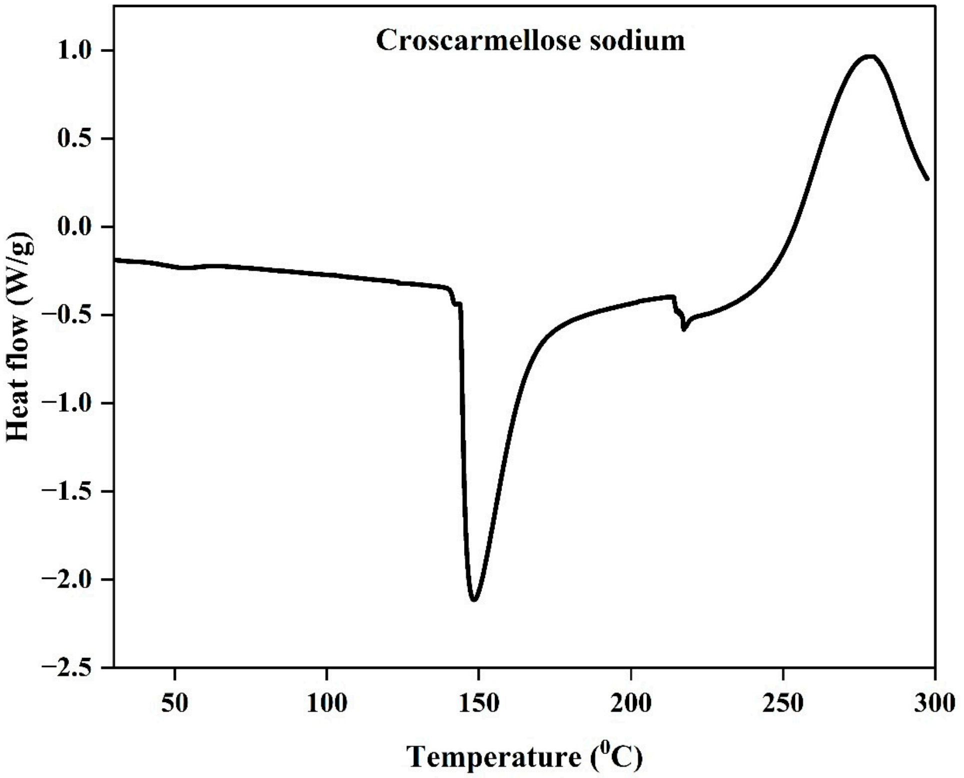
Croscarmellose sodium
Heat flow (W/g)
1.0
0.5
0.0
−0.5
−1.0
−1.5
−2.0
−2.5
50
100
150
200
250
300
Temperature (0C)

I

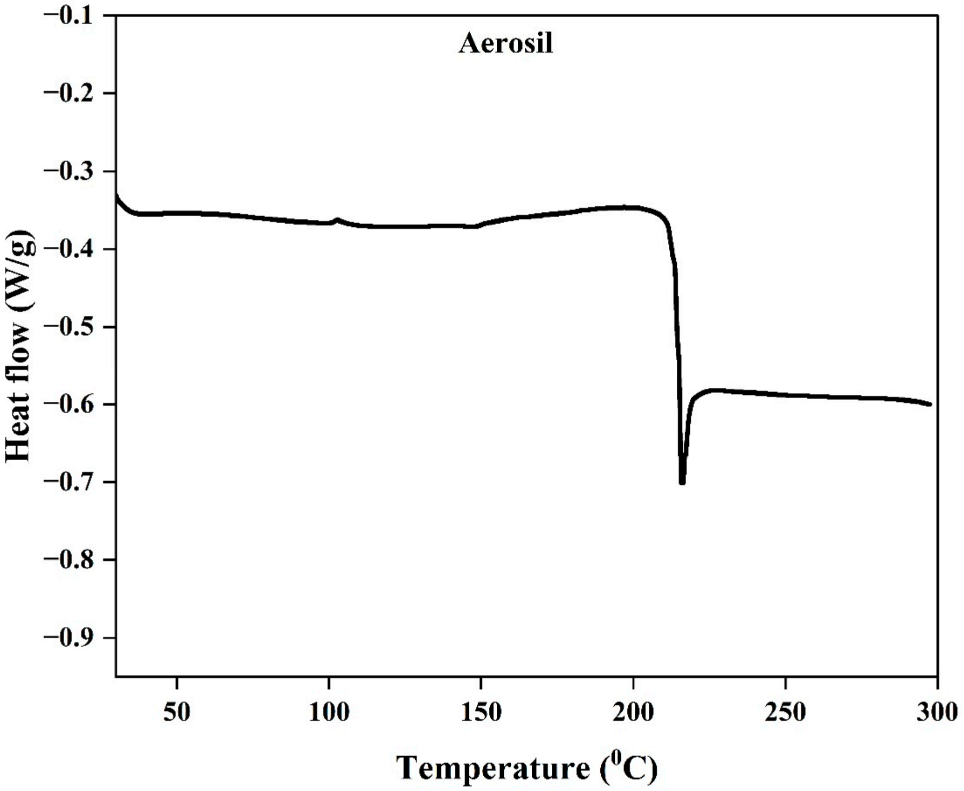

Aerosil
Heat flow (W/g)
−0.1
−0.2
−0.3
−0.4
−0.5
−0.6
−0.7
−0.8
−0.9
50
100
150
200
250
300
Temperature (0C)
